# History of myxozoan character evolution on the basis of rDNA and EF-2 data

**DOI:** 10.1186/1471-2148-10-228

**Published:** 2010-07-28

**Authors:** Ivan Fiala, Pavla Bartošová

**Affiliations:** 1 Institute of Parasitology, Biology Centre, Academy of Sciences of the Czech Republic, Branišovská 31, 370 05 České Budĕjovice, Czech Republic; 2 Faculty of Science, University of South Bohemia, Branišovská 31, 370 05 České Budĕjovice, Czech Republic

## Abstract

**Background:**

Phylogenetic relationships among myxosporeans based on ribosomal DNA data disagree with traditional taxonomic classification: a number of myxosporeans with very similar spore morphology are assigned to the same genera even though they are phylogenetically distantly related. The credibility of rDNA as a suitable marker for Myxozoa is uncertain and needs to be proved. Furthermore, we need to know the history of myxospore evolution to understand the great diversity of modern species.

**Results:**

Phylogenetic analysis of elongation factor 2 supports the ribosomal DNA-based reconstruction of myxozoan evolution. We propose that SSU rDNA is a reliable marker for inferring myxozoan relationships, even though SSU rDNA analysis markedly disagrees with the current taxonomy. The analyses of character evolution of 15 morphological and 5 bionomical characters show the evolution of individual characters and uncover the main evolutionary changes in the myxosporean spore morphology and bionomy. Most bionomical and several morphological characters were found to be congruent with the phylogeny. The summary of character analyses leads to the simulation of myxozoan ancestral morphotypes and their evolution to the current species. As such, the ancestor of all myxozoans appears to have infected the renal tubules of freshwater fish, was sphaerosporid in shape, and had a spore with polar capsules that discharged slightly sideways. After the separation of Malacosporea, the spore of the common myxosporean ancestor then changed to the typical sphaerosporid morphotype. This species inhabited the marine environment as a parasite of the gall bladder of marine fish and ultimately separated into the three main myxosporean lineages evident today. Two of these lineages re-entered the freshwater environment, one as a myxosporean with *Chloromyxum *and another with a primitive sphaerosporid morphotype. The common ancestor of all marine myxosporeans had a ceratomyxid shape of spore.

**Conclusions:**

We support rDNA based myxozoan phylogeny by the analysis of a protein coding gene and demonstrate the reliability of rDNA as a marker explaining myxozoan relationships. Our tracing the history of myxozoan character evolution discloses ancestral morphotypes and shows their development over the course of evolution. We point out several myxozoan characters that are to a certain extent congruent with the phylogeny and determined that the discrepancy between phylogeny and current taxonomy based on spore morphology is due to an extreme myxospore plasticity occurring during myxozoan evolution.

## Background

Myxozoans are microscopic metazoan parasites with extremely reduced body size and structure. Vegetative stages form spores in both hosts (invertebrates and vertebrates) in their life cycle. The only exceptions are malacosporeans in the genus *Buddenbrockia*, which live in bryozoans and have macroscopic worm-like stages in their life cycles [1]. Myxozoans were thought to be protists for more than one hundred years until the 1990 s due to the simplicity of their microscopic spores. Phylogenetic analysis of the first myxozoan SSU rDNA [2] then confirmed the marginalized suppositions that Myxozoa are multicellular organisms [3,4] and placed Myxozoans within the Metazoa. However, the phylogenetic position of the Myxozoa within the Metazoa was uncertain due to the weakness of SSU rDNA data [2,5-7]. More recent phylogenetic analysis based on the sequences of numerous protein-coding genes of the malacosporean, *B*. *plumatellae *[8], has suggested Cnidaria as the most closely related taxon to Myxozoa.

The phylogenetic analyses of an increasing number of myxosporean SSU rDNA sequences raised doubt about the taxonomic scheme of Myxosporea [9-11]. Great discrepancies were found between the phylogenetic relationships of myxosporeans inferred from the SSU rDNA and spore-based myxosporean taxonomy. A number of myxosporean species with very different types of myxospores (hence belonging to different genera) are grouped together by the phylogenetic analysis. For example, many traditional genera, such as *Henneguya*, *Sphaerospora, Myxidium*, *Zschokkella *or *Chloromyxum*, are polyphyletic. Such incongruence between taxonomy and SSU rDNA phylogeny raises questions about the reliability of the SSU rDNA as an appropriate phylogenetic marker for Myxosporea.

Generally, phylogenetic relationships based solely on a single gene reflect the phylogeny of that particular gene and may not correspond to the true species phylogeny. Moreover, single-gene analyses do not provide sufficient resolution for some nodes or sometimes give conflicting results. This is often ascribed to the limited number of nucleotides that can be aligned or to differing rates of sequence evolution leading to long-branch attraction [12,13]. Therefore, the congruent results of phylogenetic analyses based on two or more molecular markers can provide a more solid ground for better taxonomic classification. Partial sequences of the large subunit ribosomal RNA (LSU rDNA) gene have served as a second molecular marker and confirmed the SSU rDNA-based relationships of the Multivalvulida [14]. Both of these genes provided similar tree topology and were also congruent with another molecular marker (HSP-70) used at the intraspecific level in the phylogeographical study of *Kudoa thyrsites *[15]. The recent work of Bartošová et al. [16] confirmed the similar evolution of SSU and LSU rDNA genes for myxosporeans in general. The rDNA phylogenetic analyses of primary sequences were also confirmed by the analysis of variable parts of the secondary structure of SSU rDNA [17]. In the present study, we analyze elongation factor 2 (EF2) as the first protein-coding marker and we compare the obtained phylogeny to that of rDNA.

Evolution of myxozoan morphological characters became a big puzzle after the analyses of molecular data revealed the discrepancy between morphology and phylogeny. The myxospore is practically the only life cycle stage that provides a sufficient number of characters suitable for taxonomy and cladistics. Knowledge of the evolutionary history of myxospore characters is therefore important for taxonomic revisions necessary to correct myxozoan taxonomy. We constructed a matrix of morphological and bionomical characters of selected myxozoans whose rDNA sequences were available for phylogenetic analyses. Then, we mapped these characters on the SSU rDNA-based tree to trace the ancestral character features to study the evolution of myxozoan morphotypes as sets of particular character features.

## Results and Discussion

### Multigene phylogenetic analyses

**Figure 1:**
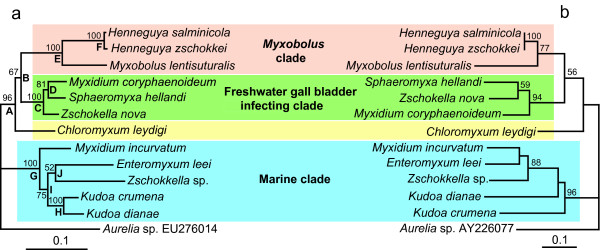
**Comparison of rDNA and EF2 based phylogeny of myxosporeans**. Maximum likelihood tree of (a) concatenated SSU + LSU rDNA data (-ln = 14993.1047, GTR + Γ model, α = 0.3415) and (b) EF2 data (-ln = 2973.9753, aa rate matrix = wag, α = 0.4692). The numbers at the nodes represent bootstrap values (> 50%). Cnidarian *Aurelia *sp. was set as outgroup sequence. Scale bar is given under the tree.

**Figure 2:**
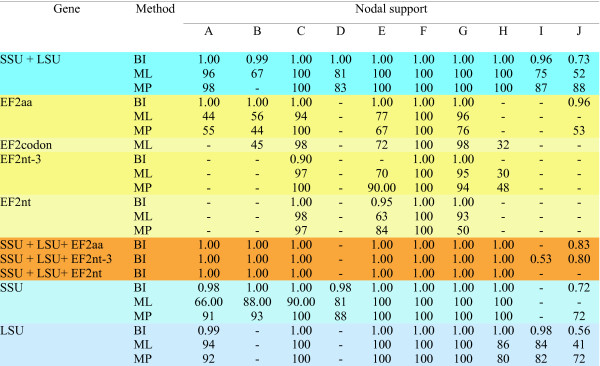
**Summary of tree nodal supports resulted from different analyses of various datasets**. Nodes A - J are specified in the Figure 1. ML = maximum likelihood; MP = maximum parsimony; BI = Bayesian inference. EF2aa = EF2 amino acid sequence data; EF2codon = EF2 sequence data analysed as codons; EF2nt-3 = EF2 nucleotide data with exclusion of the third site; EF2nt = EF2 nucleotide data. Dashes indicate missing nodes in the resulted tree.

Phylogenetic analysis of the EF2 amino acid sequence data of twelve myxosporeans (GenBank Acc. Nos. HM037908 - HM037919) was congruent with the rDNA-based phylogeny (Figure 1). Four of five nodes (ML, MP) with high bootstrap support in a concatenated SSU+LSU analysis were present in the EF2 tree. Surprisingly, the only exception was the non-sister relationship of two *Kudoa *spp. These species were also paraphyletic in additional phylogenetic analyses of nucleotide EF2 data (both with all nucleotides and excluding the third position) as well as in the analysis based on EF2 codons (additional file 1). The twelve myxosporeans studied represent three significant phylogenetic groups as defined by Fiala [11] in the SSU rDNA-based study. All of these groups were clear in our EF2 analysis. Moreover, the position of *Chloromyxum leydigi *as an independent lineage was the same. Nodes with moderate or insignificant support resulting from concatenated rDNAs analysis were frequently missing in the EF2 analyses and the sub-tree topologies were different (Figure 2). These sub-tree topologies were different when either single SSU or LSU rDNA analyses were compared to their concatenated analysis (additional file 2). These differences apply mainly for nodes in the marine clade, which includes five species representing four subgroups with unresolved relationships in broad rDNA based analysis [16]. Reasons for this incongruence may be related to the lack of informative characters that would allow resolution of particular species relationships. Concatenated analysis of all three genes (additional file 3) did not improve the nodal supports and tree resolution (Figure 2).

Congruence of rDNA and protein-coding gene analysis support the relevance of SSU rDNA as a marker for inferring myxosporean phylogeny. We can be confident that the polyphyletic and paraphyletic relationships revealed by SSU rDNA correspond to the correct species evolution. This supports the presumption that an evolutionary origin of many morphologically similar myxospore types is the result of convergent appearance of successful spore design in myxosporean evolution as pointed out for sphaerosporids by Holzer et al. [10].

### Tracing of history of myxozoan character evolution

Tracing the history of character evolution can help uncover the morphology of the ancestral myxozoan species. There are no data (and very likely there will never be) about myxozoans from the fossil record. The only possible way we can identify ancestral species is by simulating their evolution via sophisticated computer programs. We chose the Mesquite program, which is able to calculate the ancestral state of a given character by the chosen method similarly as e.g. for annelid character evolution [18]. This simulation allowed us to conceive what the ancestor of all myxozoans probably was and to find out the morphological characters of ancestors of the main clades. While the weakness of the analysis may be in the selection of available species, the general scheme of deep ancestral evolution would nevertheless not be affected.

**Figure 3:**
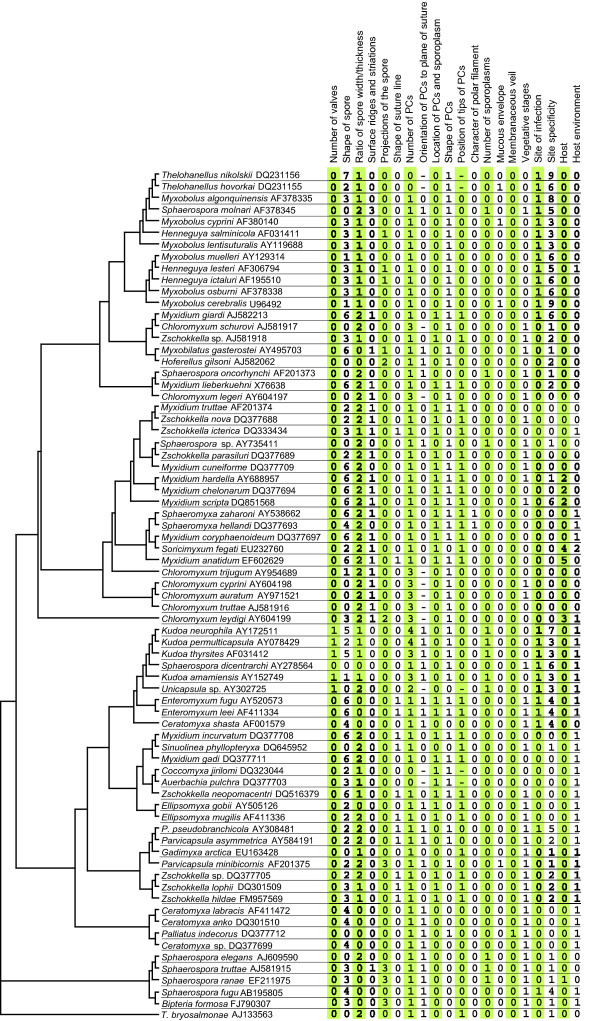
**Matrix of myxozoan characters evaluated in the analysis of character evolution**. Myxozoan ML tree based on SSU rDNA (GenBank AccNos. are behind the species names) is completed with character matrix, which is coded as follows: Number of valves: 0 - two, 1 - more than two. Shape of spore: 0 - spherical or subspherical, 1 - flattened sphere, 2 - ellipsoidal, 3 - flattened ellipsoid, 4 - banana or crescent, 4 - stellate, 5 - spindle, 6 - droplike. Ratio of dimensions of spore width to thickness: 0 - thickness larger than width, 1 - width larger than thickness, 2 - width equal to thickness. Surface ridges and striations: 0 - absent, 1 - present. Projections of the spore: 0 - no projections, 1 - caudal appendages, 2 - filamentous, 3 - bulges. Shape of suture line: 0 - straight, 1 - curved. Number of polar capsules (PCs): 0 - one, 1 - two, 2 - three, 3 - four, 4 - five and more. Orientation of PCs to a plane of the suture: 0 -PCs in the apex of the spore are set in the sutural plane, 1 - PCs are set in a plane essentially perpendicular to the suture plane. Location of PCs and sporoplasm: 0 -PCs at anterior part and sporoplasm posteriorly, 1 - PCs at opposite ends and sporoplasm in the middle. Shape of PCs: 0 - spherical or subspherical, 1 - pyriform. Position of anterior ends of PCs: 0 - convergent, 1 - divergent. Character of polar filament: 0 - tubular, 1 -flattened. Number of sporoplasms: 0 - one, 1 - two. Mucous envelope: 0 - absent, 1 - present. Membranaceous veil: 0 - absent, 1 - present. Vegetative stages: 0 -polysporic, 1 - small mono or disporic. Site of infection: 0 - coelozoic, 1 - histozoic. Site specificity: 0 - gall bladder and biliary ducts, 1 - renal tubules, 2 - urinary bladder, 3 - muscle, 4 - intestine, 5 - gills or pseudobranchs, 6 - without site specificity, 7 - nervous system, 8 - ovary, 9 - cartilage. Host: 0 - fish, 1 - amphibian, 2 - reptile, 3 - elasmobranchs, 4 - mammal, 5 - bird. Host environment: 0 - freshwater, 1 - marine, 2 - terrestrial.

**Figure 4:**
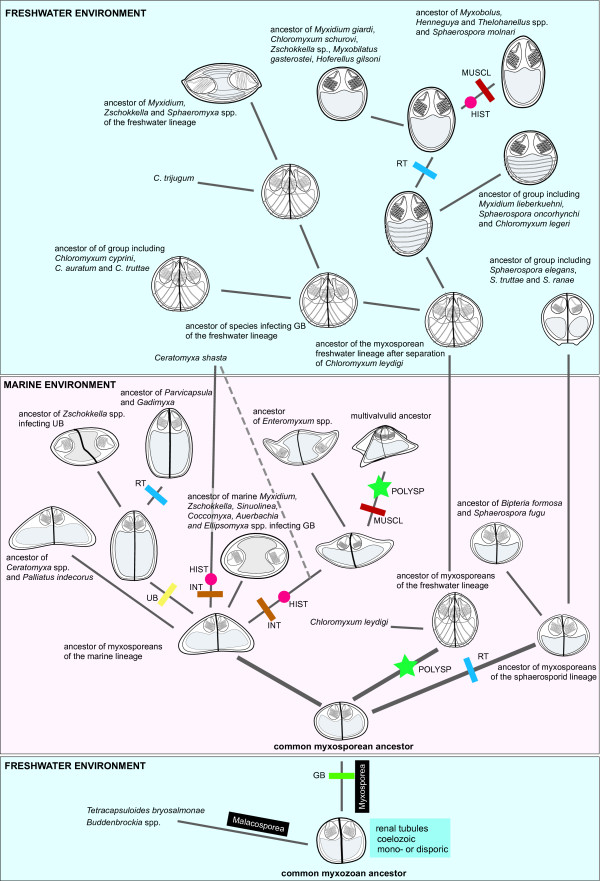
**Hypothetical evolution of ancestral morphotypes and their bionomical characters**. Schematic drawing summarises evolution of myxospore morphology based on the SSU rDNA phylogeny and character evolution analysis. Rectangles, circles and asterisks show the switches of bionomical characters. GB - gall bladder, UB - urinary bladder, RT - renal tubules, MUSCL - muscles, INT - intestine, HIST - histozoic, POLYSP - polysporic. Dashed lines indicate two alternative branches leading to *Ceratomyxa shasta*.

We mapped 15 morphological and 5 bionomical characters on the SSU rDNA tree, which was constructed to cover all known phylogenetic groups of myxosporeans. The matrix includes also five unnamed, formally not characterized species, since they represent significant morphotypes and phylogenetically important taxa for the clades in which these species cluster. The summary of all characters, features, and taxa used in this analysis is shown in Figure 3. We inferred a character history for each character using likelihood ancestral state reconstruction methods to understand the process of individual character change as well as the change of the spore as a complex of characters. Figure 4 summarizes the evolutionary history of myxospore morphology and the presumed switches among bionomical characters based upon this analysis. Cladograms in additional file 4 illustrate the particular character histories for all determined characters.

#### Number of valves

The ancestor of all myxozoans had two spore valves. The analysis showed that the change to the two-valve character state evolved only once in an ancestor in one of the marine lineages. The number of valves is the main taxonomic characteristic on which the separation of the class Myxosporea into the orders Bivalvulida Shulman, 1959 and Multivalvulida Shulman, 1959 is based. Evolution of this character makes Bivalvulida a paraphyletic group and renders the basic taxonomic division of Myxosporea erroneous.

#### Shape of spore

Myxosporeans have a variable spore shape that is difficult to delimitate into a few defined categories. We divided myxospores into eight basic three-dimensional categories for the purpose of our analysis (See legend in additional file 4b). The common myxozoan ancestor had a spherical spore. The myxospore changed its shape by the enlargement of its width to a crescent or banana-like shape after the switch to parasitizing marine fish. Descendants of this marine ancestor then diversified into several different spore shapes, which is in contrast to the rather uniform spore shape in ancestors of the main groups of the freshwater lineage.

#### Ratio of dimensions of spore width to thickness

This character is derived from the shape of the spore and uses only three features to characterize spore dimensions. Such a simple definition of spore shape facilitated the tracing of the evolution of spore shape and showed that the ratio of spore dimensions is a useful character shared in many phylogenetic groups. The analysis suggested that the ancient ancestors had spores thicker or of the same thickness as the width. Spores wider than thick appeared more recently in the myxosporean evolution.

#### Surface ridges and striations

The analysis showed that the primitive feature of this character is a smooth shell valve. Surface ridges then evolved in an ancestor of the freshwater lineage after the separation of myxosporeans into marine and freshwater lineages. Surface ridges are absent in all marine species except for several marine species clustering out of the marine clade, e.g. *Myxidium coryphaenoideum*, *Sphaeromyxa zaharoni *and *Chloromyxum leydigi*. This supports the position of *Sphaeromyxa*, a typical marine genus with a history of parasitism in the freshwater fishes, inside the freshwater lineage.

#### Projections of the spore

A typical example of spore projections are the caudal appendages on *Henneguya *spores. Species with this feature, distinguishing *Henneguya *from *Myxobolus *spp., do not share a common ancestor. Our analysis includes only several *Myxobolus *and *Henneguya *sequences, which does not reflect the true multiple origin of caudal appendages and the polyphyletic pattern of *Henneguya *spp. as described elsewhere [9,11]. Spore filaments are less frequent features that independently appeared only in the two terminal taxa under study. Spore bulges evolved in the ancestor of basal freshwater sphaerosporids and independently in *Bipteria formosa*, *Parvicapsula minibicornis *and *Sphaerospora molnari*.

#### Shape of suture line

A curved sutural line is a typical homoplastic character found several times on terminal nodes of certain *Myxidium*, *Zschokkella*, *Parvicapsula*, *Enteromyxum *and *Sinuolinea *spp.

#### Number of polar capsules

The ancestral state for the number of polar capsules (PCs) is two. After the split of Myxozoa into the two major freshwater and marine clades, the ancestor of the lineage with mostly freshwater species probably multiplied its PCs to four. A four-PC spore was then reduced twice to the two-PC spore within the evolution of this lineage. The four-PC spore then appeared independently again in the terminal nodes represented by *Chloromyxum legeri *and *C*. *schurovi*. Multiplication of PCs in the multivalvulids (*Kudoa *spp. and *Unicapsula *sp.) evolved along with an increase in number of spore valves. The two-PC character of spore changed twice to a spore with only one PC in myxozoan evolution: 1) from the ancestor having two PCs at opposite ends (in the marine lineage) and evolving to *Auerbachia *and *Coccomyxa *spp.; and 2) from the ancestor having its PCs at the anterior end (in the freshwater lineage) and evolving to *Thelohanellus *spp.

#### Orientation of polar capsules to a plane of the suture

The ancestor of all myxozoans had the PCs arranged in the plane perpendicular to the suture line. The ancestor of species of the freshwater lineage changed the position of the PCs and the primitive state reappeared independently several times in *Sphaerospora *spp., *Hoferellus gilsoni *and *Myxobilatus gasterostei*. The opposite situation can be seen in the marine clade: ancestors of several clades possessed primitive characters and the PC arrangement to a plane of the suture evolved in the recent ancestors of current species.

#### Location of polar capsules and sporoplasm

The primitive state is the position with PCs together at the anterior part of the spore with a posterior sporoplasm. PCs moved to the opposite end of the spore quite recently in evolution. This character evolved independently several times and it is common for species of four distinct phylogenetic groups including species of genera e.g. *Myxidium*, *Zschokkella *and *Enteromyxum*.

#### Shape of polar capsules

The ancestor of all myxozoans had spherical or subspherical shape of PCs. Pyriform PCs evolved in the ancestors of the freshwater branch. Further, pyriform PCs appeared independently several times within the marine lineage.

#### Position of tips of polar capsules

The evolution of this character is linked to the arrangement of PCs within a spore. Divergent PCs evolved in parallel with the evolution of PCs in opposite positions and the history of their evolution is similar. One of few exceptions of this "co-evolution" is *Tetracapsuloides bryosalmonae *possessing divergent PCs arranged together in its fish malacospore.

#### Character of polar filament

The feature of having flattened polar filaments evolved only once and it is unique to species of the genus *Sphaeromyxa*.

#### Number of sporoplasms

The ancestral state is a single sporoplasm. A spore with two sporoplasms appeared in the common ancestor of basal sphaerosporids and multivalvulids and in several current *Sphaerospora *spp.

#### Mucous envelope and membranaceous veil

Most of myxozoans do not possess any of these spore characters. The presence of a mucous envelope or membranaceous veil appeared independently only a few times in terminal nodes and has no common evolutionary history.

#### Vegetative stages

The common ancestors of all myxozoans and the ancestor of species clustering in the marine lineage had small mono- or disporic plasmodia. Polysporic plasmodia probably evolved for the first time in the ancestors of the freshwater lineage and then twice independently in the marine lineage, whereas the reverse reduction of large plasmodia to the smaller ones occurred probably many times.

#### Site of infection

We analysed the site of infection as a two-state character defined as coelozoic and histozoic. It seems that there were only two main splits from coelozoic to histozoic site preference in the evolution of Myxozoa leading to: 1) *Myxobolus*/*Henneguya *species and 2) to the group containing multivalvulids and enteromyxids (including *Ceratomyxa shasta *in some analyses).

#### Site specificity

This multi-state character extends the previous one by more specific definition of the site of infection. The analysis of a particular site of infection suggested that the first myxozoans inhabited the excretory system. Later myxozoans then infected the gall bladder. The ancestors of several groups moved back from the gall bladder to the excretory system, intestine, or the muscles. Only the histozoic *Myxobolus *and *Henneguya *spp. show great variability in the site of infection. Many other phylogenetic groups include species with identical site specificities.

#### Host

The typical intermediate host of myxozoans is a fish. It is very likely that the ancestors of the main myxosporean groups infected the fish whereas several switches to other vertebrate taxa occurred later.

#### Host environment

The first myxozoan ancestor was a freshwater species and then myxozoans expanded to the marine environment. Thus, the ancestor of all Myxosporea was a marine species. Freshwater as the host environment occurred three times independently: by the ancestor of species of the freshwater lineage, by the ancestor of the freshwater basal sphaerosporid, and by *Ceratomyxa shasta*. Some freshwater species then re-entered the marine environment afterward. A typical returnee was the ancestor of species of the genus *Sphaeromyxa*, a diversified group of marine myxosporeans.

Tracing the evolution of particular characters facilitated the reconstruction of the evolution of the myxospores' morphology and bionomical characters (Figure 4). Based on our analyses, the ancestor of all myxozoans had a spore with two valves, was spherical in shape, had a width that was equal to its thickness, had no surface ridges and no projections, and had a straight suture line. Further, the spore had two subspherical and convergent PCs set in the plane perpendicular to the suture, with the PCs arranged at its anterior end. There was one posterior sporoplasm, a tubular polar filament, and no mucous envelope or membranaceous veil. The plasmodia were small and mono- or disporic, the site of infection was coelozoic, and it was a parasite of the excretory system of freshwater fishes. Furthermore, our analyses suggested some common trends in the myxosporean character evolution e.g.: i) myxosporeans with four PCs and two shell valves evolved only in the freshwater clade; ii) myxosporeans with four or more shell valves evolved only once in the marine clade; iii) myxosporeans with surface ridges or striations and caudal appendages evolved only in the freshwater clade; and iv) myxosporeans with PCs placed on opposite sides of the spindle or ellipsoid shaped spore evolved several times in both marine and freshwater clades. We found only one clear morphological synapomorphic character following the phylogenetic relationships of myxozoans. Although such incongruence between phylogeny and taxonomy is extreme among eukaryotes, a similar situation was documented in the Microsporidia [19]. Species of this fungal group reduced their body form to the plasmodial and spore stages similarly to the myxozoans. Further, and also similar to what occurs with the Myxozoa, the Microsporidian taxonomy based on the morphology of the spore is not congruent with the phylogeny of the group. The sole synapomorphic character is the polar filament. Its arrangement and flattened shape is unique for *Sphaeromyxa *spp. and it is one of the most important characters for the definition of the genus *Sphaeromyxa*. The other 19 characters that we traced were homoplastic. Nevertheless, bionomical characters (site of infection, site specificity, and host environment) correlate highly with the phylogeny. Although the myxospore's morphology generally does not correlate with the phylogeny, several morphological characters were found to correspond partially to the phylogeny. These myxospores characters are: the number of valves, the position of PCs relative to the sutural plane and the sporoplasm, the ratio of spore width to thickness, and the presence or absence of surface ridges. This is illustrated by the frequent presence of clusters of species with the same character features in the phylogenetic trees (additional file 4). Connection of these characters could be utilised in future taxonomic changes (taxon demises or the establishment of new genera or higher taxonomic levels) together with the combination of bionomical characters.

The main phylogenetic segregation according to the myxozoans hosts' habitat (freshwater and marine) were firstly documented by Kent et al. [20] and this trend is, in general, still valid with some exceptions. Our analyses also supported the site of infection and site specificity as unique characters that follow the phylogenetic pattern of many myxozoan species as published elsewhere [21,10,22,11]. However, there are some exceptions to this trend (e.g., *Sinuolinea phylopteryxa*, *Myxidium hardella *or *Sphaerospora *sp. EE2004). These latter parasites of renal tubules cluster among the gall bladder infecting species. Nevertheless, site specificity is still a common feature for many closely related species. Tracing the history of evolution of this character enables the assignment of the gall bladder site as a feature that evolved early in evolution. This feature persists among descendants and occurs among many current species. This means that all gall bladder infecting species with known SSU rDNA have ancestors with gall bladder site specificity except for the deepest ancestor of all myxozoans, which infected the excretory system. Furthermore, all species with different site specificity than the gall bladder evolved from ancestors infecting the gall bladder. This corresponds to Shulman's assumption [23] that the first myxosporean species was coelozoic and infected the gall bladder.

Spore projections or surface ridges can be considered as typical examples of homoplastic characters. They very likely have identical biological function and resulted from the convergent evolution in myxosporean species. Ridges probably help spores to float in freshwater, an assumption that is supported by the absence of such features in species from the marine environment, whose water has a higher density. The exception is the marine species *Chloromyxum leydigi*, which has a close phylogenetic relationship to the freshwater lineage. Surprisingly, no ridges appeared on the typical freshwater species-rich genus *Myxobolus*. However, floating in this case is probably facilitated by the development of caudal appendages. This character evolved independently many times during the diversification of numerous *Myxobolus *spp. and gave rise to the current *Henneguya *spp. Caudal appendages similar to those found in *Henneguya *spp. are also present in species of the genera *Unicauda*, *Dicauda, Hennegoides*, *Tetrauronema*, and *Laterocaudata*. Although the SSU rDNA sequences of these species are not available in GenBank, they would probably cluster within the *Myxobolus *clade and would be expected to have a similar evolutionary history as that of *Henneguya *spp.

Based upon our analysis caudal appendages should be considered as species characteristics but not generic characters. Suppression of the genus *Henneguya *can be expected as well as suppression of the genera *Hennegoides*, *Tetrauronema*, *Laterocaudata*, *Dicauda *and *Unicauda *after molecular data is obtained. Some taxonomic revisions, resulting from phylogenetic analyses, have been already carried out. Whipps et al. demised the genera *Pentacapsula*, *Hexacapsula*, and *Septemcapsula *and assigned their species to the genus *Kudoa *[14]. Further, *Zschokkella mugilis *was transferred to the genus *Ellipsomyxa *[24]. A radical taxonomic revision was also recently made by Gunter and Adlard, who demised the genus *Leptotheca *and transferred its species to the genera *Ceratomyxa *and *Sphaerospora *[25].

### Evolution of myxozoan morphotypes

Our analysis has shown that the myxozoan common ancestor was a species with a spore morphology similar to that of current species of the genus *Sphaerospora *infecting the renal tubules of freshwater fishes. This supports the hypothesis of Jirků et al. that current myxozoans are derived from the *Sphaerospora*-type myxospore [26]. We confirmed that a group of basal sphaerosporids possesses features ancestral to present species. On the other hand, some *Sphaerospora *spp. with known SSU rDNA sequences branched at several distinct positions in the tree, which could be explained by a certain plasticity in the change of myxospores' shape that would best fit environmental factors. This supports the idea that the sphaerosporid morphotype might be a very successful spore design favoured by convergent evolution [10].

**Figure 5:**
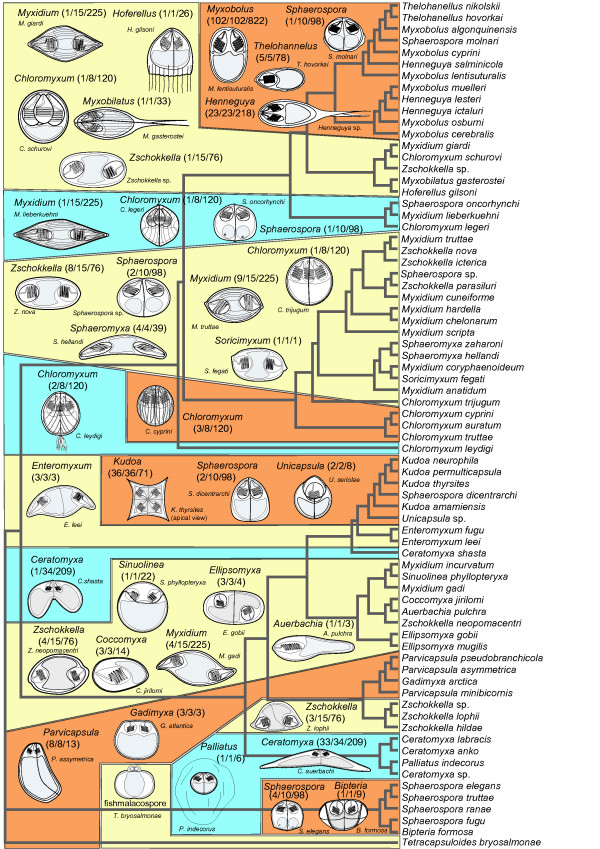
**Spore morphology of selected myxozoans of all genera included in the main myxozoan phylogenetic groups**. Maximum likelihood SSU rDNA tree of selected myxozoans covering the broad phylogeny of Myxozoa. The boxes join the myxozoans of main phylogenetic groups. Each box contains schematic drawings of the spores of representatives of all genera included in the group. Numbers in the brackets by the genera names show the number of sequenced species of the genus placed in the clade/number of overall sequenced species of the genus/number of described species of the genus.

Based on our results, we propose several major trends in the evolution of myxospore morphotypes (Figure 4). The morphotype means a set of spore morphological characters typical for group of myxosporeans assigned to the same myxosporean genus (e.g. *Myxidium *or *Myxobolus *morphotype). According to Lom and Dyková there are about 60 described genera of Myxosporea [27], which ergo represent the same number of current spore morphotypes. A hypothetical ancestor of all species in the marine clade evolved from the ancestral sphaerosporid morphotype by an extension of the spore thickness, giving rise to the ceratomyxid morphotype. This morphotype is retained through the present as a group of species belonging to the genus *Ceratomyxa*. Intestinal *Ceratomyxa shasta *may have evolved either from a gall bladder infecting ancestral ceratomyxid (regardless of its uncertain phylogenetic position as a separate lineage in the marine clade [11]) or as a basal ceratomyxid [25]. We suggest a different hypothesis, that *C*. *shasta *shared a common ancestor with both enteromyxids and multivalvulids. After the switch from parasitism in the gall bladder to a site location in the intestine, *C*. *shasta *then separated from the enteromyxid and multivalvulid ancestor (dashed line in Figure 4). This possibility is the most parsimonious solution, with only one switch to different site of infection. This theory is also supported by our ML analysis (Figure 5). However, the analysis includes the limited number of *Ceratomyxa *sequences. The sphaerosporid morphotype very likely occurred by the ancestor of *Parvicapsula *spp. infecting the excretory system. Spores of *Parvicapsula *spp. are very similar to those of *Sphaerospora *spp. and basically represent a further step in the evolution of the sphaerosporid type of spore. The kudoid morphotype probably evolved by a duplication or multiplication of an ancestral extinct morphotype with an enteromyxid spore shape but with PCs close together. The PCs then independently moved to the opposite ends of the spore along with an extension of spore width several times in myxosporean evolution. This evolution gave rise to the species classified to the genera *Myxidium*, *Zschokkella*, *Ellipsomyxa, Sphaeromyxa *and *Enteromyxum*. Intestinal *Enteromyxum *spp. very likely did not evolved from the *Myxidium *or *Zschokkella *morphotype. Despite some similarities in the spore structure with *Myxidium *morphotype, *Enteromyxum *spp. probably evolved from the gall bladder infecting ceratomyxid ancestor. The morphotype with four polar capsules and two shell valves similar to that of existing *Chloromyxum *spp. probably evolved early after the separation of the marine lineage from the rest of the myxosporeans, including the freshwater lineage and marine *Chloromyxum leydigi*. We can hypothesize that the *Chloromyxum *morphotype was derived from an ancestral sphaerosporid by the duplication of polar capsules. Later in the evolution, myxospores then lost these additional two polar capsules, creating the predominant *Myxobolus*, *Zschokkella *and *Myxidium *morphotypes of the freshwater clade. *Sphaeromyxa *species had evolved by a change in the character of polar filament from the *Myxidium *ancestor, which then moved back to the marine environment. Members of the most species-rich myxozoan genus, *Myxobolus*, probably evolved from the coelozoic ancestor infecting renal tubules. *Myxobolus *spp. invaded numerous host tissues and then segregated to numerous species during evolution. The origin of caudal appendages present on the *Myxobolus *morphotype led to the evolution of species classified in the genus *Henneguya*. *Thelohanellus *spp. evolved from the *Myxobolus *morphotype by the loss of one of its PCs. The same event probably occurred in both *Auerbachia *and *Coccomyxa *spp., which arose from the *Myxidium *morphotype in the marine clade. The origin of *Myxobilatus *and *Hoferellus *morphotypes with PCs placed perpendicular to the suture plane is another proof that the polar capsules freely change their position relative to the suture plane during evolution. This is typical for the origin of *Sphaerospora *species, which evolved convergently several times in the marine or freshwater clade. *Gadimyxa *spp. also differ from their parvicapsulid ancestor in the arrangement of PCs to a plane of the suture.

### Character evolution and phylogeny

A summary of the spore morphology of selected myxosporeans of all genera of the main myxozoan phylogenetic groups is presented in the Figure 5. This summary demonstrates spore variability throughout the myxozoan phylogenetic tree, as well as the close relationship of species with very different spore structure and, vice versa, the distant relationships of species with very similar spore morphology. Figure 5 also gives the proportion of sequenced species (SSU rDNA) of each genus present in the particular clade relative to the number of all sequenced species of each genus and to overall number of described species of each genus. These numbers illustrate the lack of data in many genera. Ten out of about one hundred known species of *Sphaerospora *cluster into five different positions on the SSU rDNA tree. Holzer et al. interpreted this phenomenon to be a result of the convergent evolution of the primitive sphaerosporid spore shape [10]. The phylogenetic position of ninety *Sphaerospora *spp. is still not known. The extreme polyphyletic nature of species rich genera, such as *Sphaerospora*, *Myxidium*, *Zschokkella *and *Chloromyxum*, suggests that the many myxosporeans that have not yet been sequenced likely may cluster in any phylogenetic branch of the tree.

Significantly, molecular rDNA data are not able to resolve several evolutionary important nodes in the myxosporean phylogenetic tree [16]. The relationships of the main myxosporean phylogenetic groups of the marine clade, as defined in Fiala [11], are uncertain and depend on taxa sampling and the analysis performed. Tracing the evolutionary history of studied characters may enable resolution of some uncertain relationships. It has been proven that site of infection is a factor connecting many myxosporeans. Both *Ceratomyxa shasta *and *Enteromyxum *spp. have uncertain position in the marine clade with low bootstrap supports [11] and identical sites of infection suggest their common evolution. The spore morphology of the hypothetical ancestor of enteromyxids and multivalvulids is likely very similar to *C*. *shasta*. However, this disagrees with a recent analysis of a large number of *Ceratomyxa *spp., wherein *C*. *shasta *branches as a basal taxon to all ceratomyxid species [28]. Nevertheless, *Kudoa *spp. + *Enteromyxum *spp. and *C*. *shasta *may constitute one histozoic clade in myxosporean evolution.

## Conclusions

In this study we support rDNA based myxozoan phylogeny by sequencing and analyses of the protein coding gene EF2 and demonstrate the reliability of rDNA as a marker explaining myxozoan relationships. We propose the evolution of ancestral myxozoan morphotypes. The evolutionary history enables us to understand the evolution of modern species and supports some uncertain topologies resulting from the analyses of SSU rDNA data.

## Methods

### DNA isolation, PCR amplification, cloning and sequencing

DNA was extracted from fresh myxospores using the DNeasy™Tissue Kit (Qiagen, Germany) according to the manufacturer's protocol. EF2 gene was amplified using A1 (5'-GGNGCNGGNGARYTNCAYYTNGA-3) and A2 (5'-CCARTGRTCRAANACRCAYTGNGGRAA-3) primer set [29]. PCR was carried out in a 25 μl reaction volume using 10 pmol of each primer, 250 μM of each dNTP, and 2.5 μl 10 × PCR Buffer (Top-Bio, Czech Republic) and 1 unit of Taq-Purple polymerase (Top-Bio, Czech Republic). The reactions were run on a Tpersonal cycler (Biometra). Amplification consisted of initial denaturation at 95°C for 5 min and 35 cycles of 95°C for 1 min, 48°C for 1 min and 72°C for 1 min, followed by 10 min final extension at 72°C. The PCR products were isolated from the gel and cloned into pCR^® ^2.1-TOPO vector from the pDrive Cloning vector (Qiagen PCR Cloning Kit) and transformed into competent *E*. *coli*-strain XL-1. Both strands of clones were sequenced on ABI PRISM 3130xl automatic sequencer (Applied Biosystems).

### Alignments and phylogenetic analyses

Seven alignments of EF2, SSU and LSU data with twelve myxosporeans were constructed. The cnidarian *Aurelia *sp. was set as outgroup. The alignments consisted of single gene data, concatenated SSU + LSU and concatenated SSU + LSU + EF2 data. EF2 gene was aligned as nucleotide sequence (EF2nt) as well as amino acid (EF2aa) sequence data. EF2nt were analysed with all three sites in codon, with the exclusion of the third site of codon and as codons. The alignments were aligned using MAFFT program [30] with E-INS-i method and gap opening penalty (--op) 5.0 and gap extension penalty (--ep) 0.0. The alignments were visualised in SEAVIEW v. 3.2 [31].

Maximum parsimony (MP) analyses were performed in PAUP* 4.0b10 [32] using a starting tree build by heuristic search with random taxa addition, the ACCTRAN-option and the TBR swapping algorithm. Ts/Tv ratio of 1:2 was applied for rDNA data and the gaps were treated as missing data. Clade supports were estimated using 500 bootstrap-replicates with random sequence additions.

Maximum likelihood (ML) analyses of EF2aa and EF2nt as codon sequence type were performed in GARLI v. 0.96 [33]. Amino acid analysis was done using WAG model and F3x4 method of codon frequencies was chosen for the codon based analysis. All the other ML analyses were made in PAUP* 4.0b10. The best model of evolution was determined by the likelihood ratio test (LRT) implemented in the Modeltest 3.06 [34]. SSU, LSU and SSU + LSU analyses were performed under GTR + Γ model. EF2nt analysis was performed under GTR + Γ + I model. Bootstrap values were calculated by 500 bootstrap re-sampling.

Bayesian inference (BI) trees were constructed using MrBayes 3.0b4 [35]. Likelihood parameters that were set for the single gene analyses and for the data partitions of concatenated analyses correspond to the models used in ML. The number of Markov chain Monte Carlo (MCMC) generations was set to 1,000,000 with every 100th tree saved (two independent runs of four simultaneous MCMC chains). AWTY system [36] was used to assess the length of MCMC run and Tracer v. 1.4.1 [37] was used to ascertain the length of burn-in periods.

### Morphological matrix and mapping of morphological characters

Twenty morphological and bionomical characters were chosen for the analysis (Figure 3). The analysis includes (1) number of spore valves, (2) shape of spore, (3) ratio of dimensions of spore width to the thickness, (4) surface ridges and striations, (5) projections of the spore, (6) shape of suture line, (7) number of polar capsules, (8) orientation of polar capsules to a plane of suture, (9) location of polar capsules and sporoplasm, (10) shape of polar capsules, (11) position of tips of polar capsules, (12) character of polar filament, (13) number of sporoplasms, (14) mucous envelope, (15) membranaceous veil, (16) vegetative stages, (17) site of infection, (18) site specificity, (19) host, and (20) host environment. The SSU rDNA-based tree was chosen as the basis for reconstruction of ancestral states. The tree consists of 73 myxosporean species covering the myxosporean diversity of known SSU rDNA sequences and malacosporean *Tetracapsuloides bryosalmonae *as the outgroup. The tree was constructed using ML with GTR + Γ + I model of evolution in PAUP* based on the alignment computed by MAFFT with parameters described above. Tree branches with uncertain phylogenetic relationships - unstable positions in the tree as described in Fiala [11] - were collapsed in their nodes resulting into polytomy for more accurate reconstruction of myxozoan phylogenetic relationships. History of character change was traced using the program Mesquite 2.5 [38]. Reconstruction of character states at ancestral nodes was done by likelihood method. We used Markov k-state 1 parameter model with the single parameter (the rate of change) [39]. Any particular change from one state to another is equally probable within this model.

## Authors' contributions

IF carried out the DNA sequence studies, performed phylogenetic analyses, performed the analyses of character evolution and drafted the manuscript. PB participated on DNA sequenced studies, participated on compiling of character matrix and helped to draft the manuscript. Both authors approved the final manuscript.

## Supplementary Material

Additional file 1 **Alternative EF2 tree topologies**. Maximum likelihood tree topologies of a) EF2 nucleotide sequence based analysis (-ln = 6943.4742, GTR + Γ + I model, α = 1.0804, pinvar = 0.2586), b) EF2 nucleotide sequence based analysis with exclusion of the third site of codon (-ln = 3280.0776, GTR + Γ + I model, α = 1.6415, pinvar = 0.4103), c) EF2 codon based analysis (-ln = 6513.1449, state frequencies f3x4 method). Bootstrap values (> 50%) are indicated at the nodes. Cnidarian *Aurelia *sp. was set as outgroup sequence. Scale bar is given under the tree. Click here for file

Additional file 2 **SSU and LSU rDNA tree topologies**. Maximum likelihood trees constructed under a) SSU rDNA data (- ln = 6127.1469, GTR + Γ model, α = 0.3861) and b) LSU rDNA data (- ln = 8824.2894, GTR + Γ model, α = 0.317). Bootstrap values (> 50%) are indicated at the nodes. Cnidarian *Aurelia *sp. was set as outgroup sequence. Scale bar is given under the tree. Click here for file

Additional file 3 **Combined SSU, LSU and EF2 tree topologies**. The phylogenetic trees based on Bayesian inference of combined SSU rDNA + LSU rDNA + EF2 data. a) EF2 as amino acid sequence data, b) EF2 as nucleotide sequence data, c) EF2 as nucleotide data with exclusion of the third site of codon. The Bayesian posterior probabilities are indicated at the nodes. Cnidarian *Aurelia *sp. was set as outgroup sequence. Scale bar is given under the tree. Click here for file

Additional file 4 **Evolution of particular myxozoan characters**. Twenty cladograms show the evolutionary history of all morphological and bionomical characters under study. The balls by the nodes represent proportional likelihoods of character states. See legends for colours of particular character states. Click here for file
