# Supplementary figures and images for: History of myxozoan character evolution on the basis of rDNA and EF-2 data
Source: BMC Evol Biol. 2010 Jul 28;10:228. doi: 10.1186/1471-2148-10-228 (PMC2927925; doi:10.1186/1471-2148-10-228)

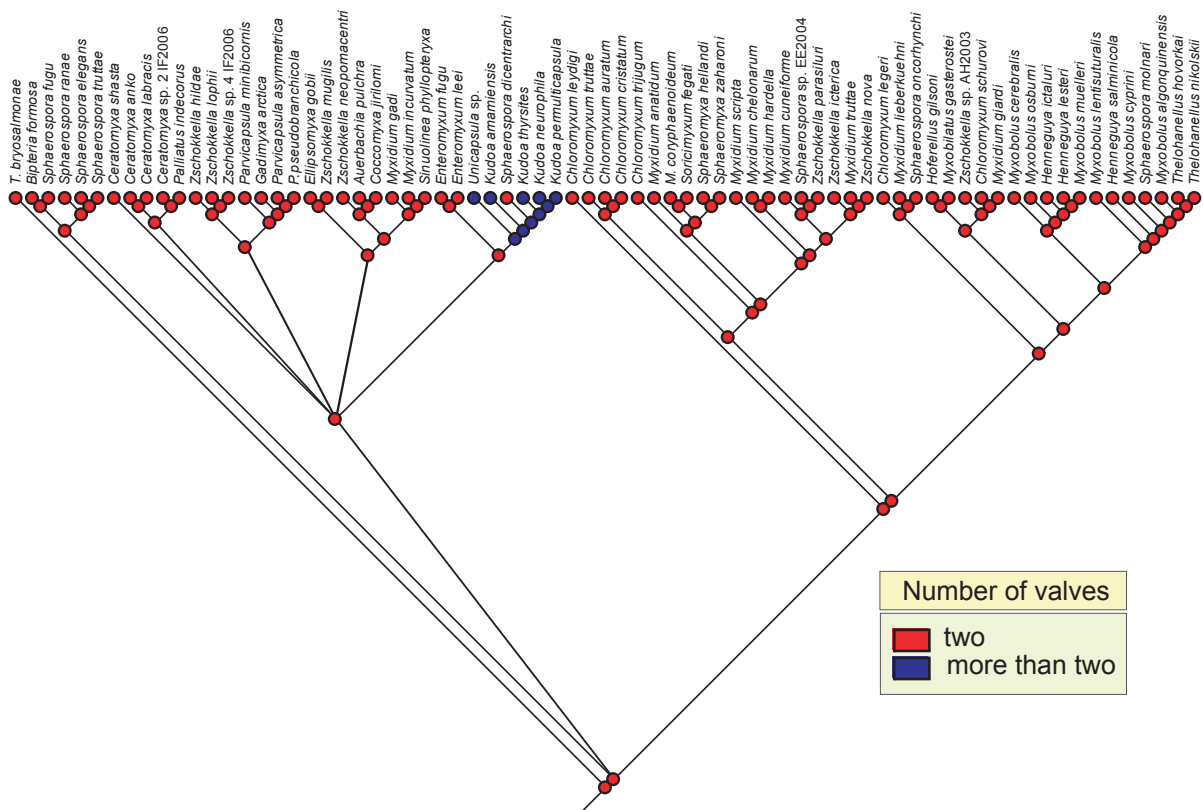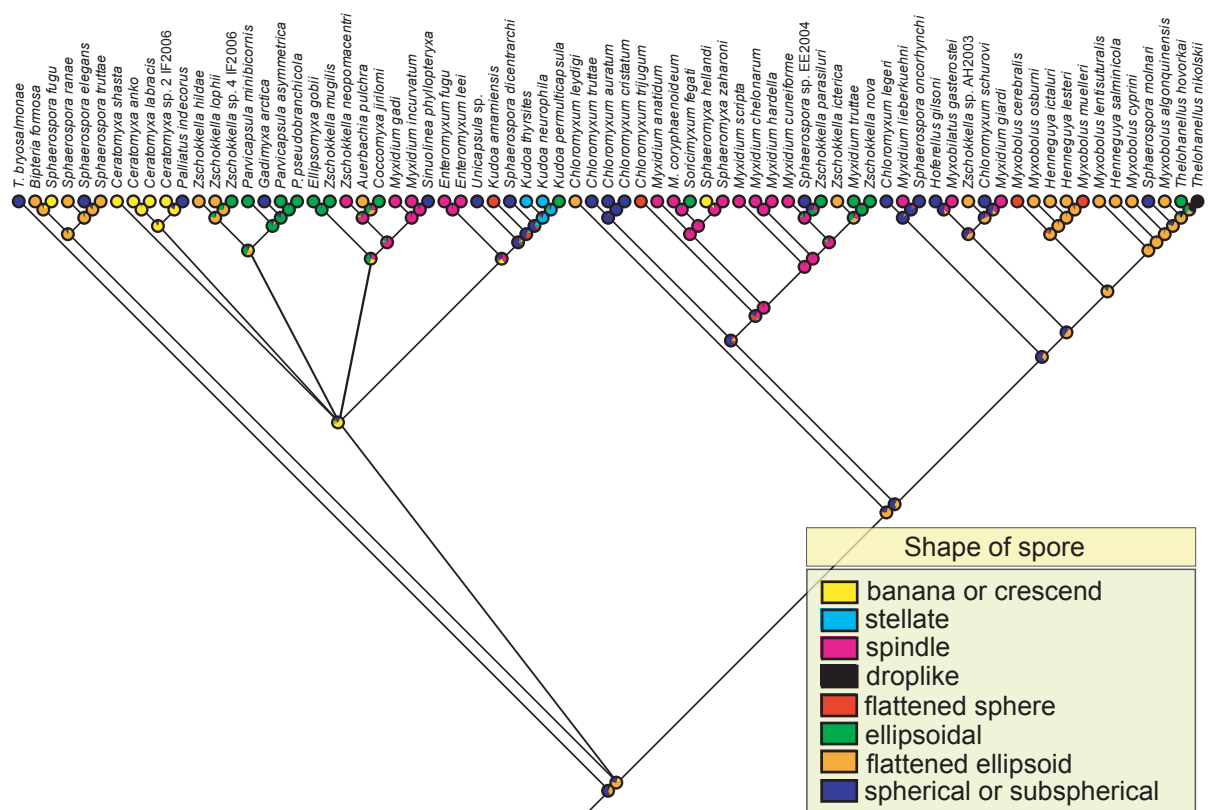

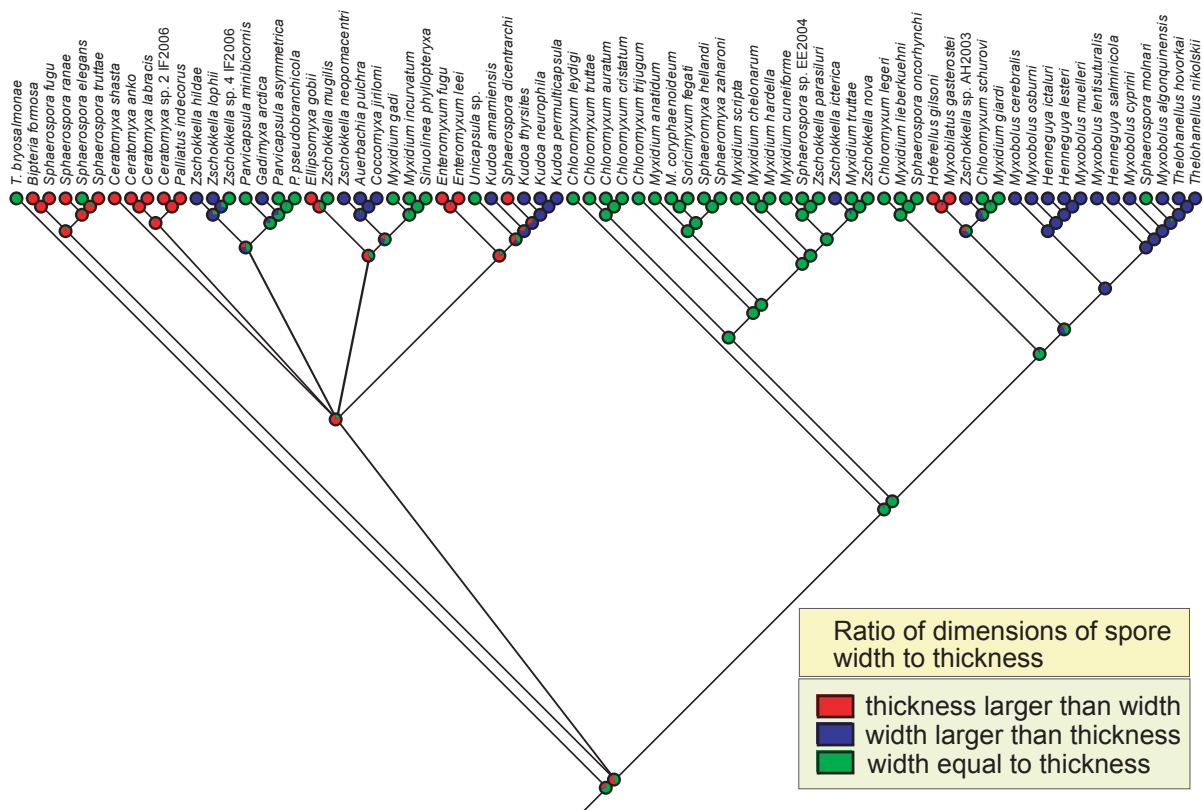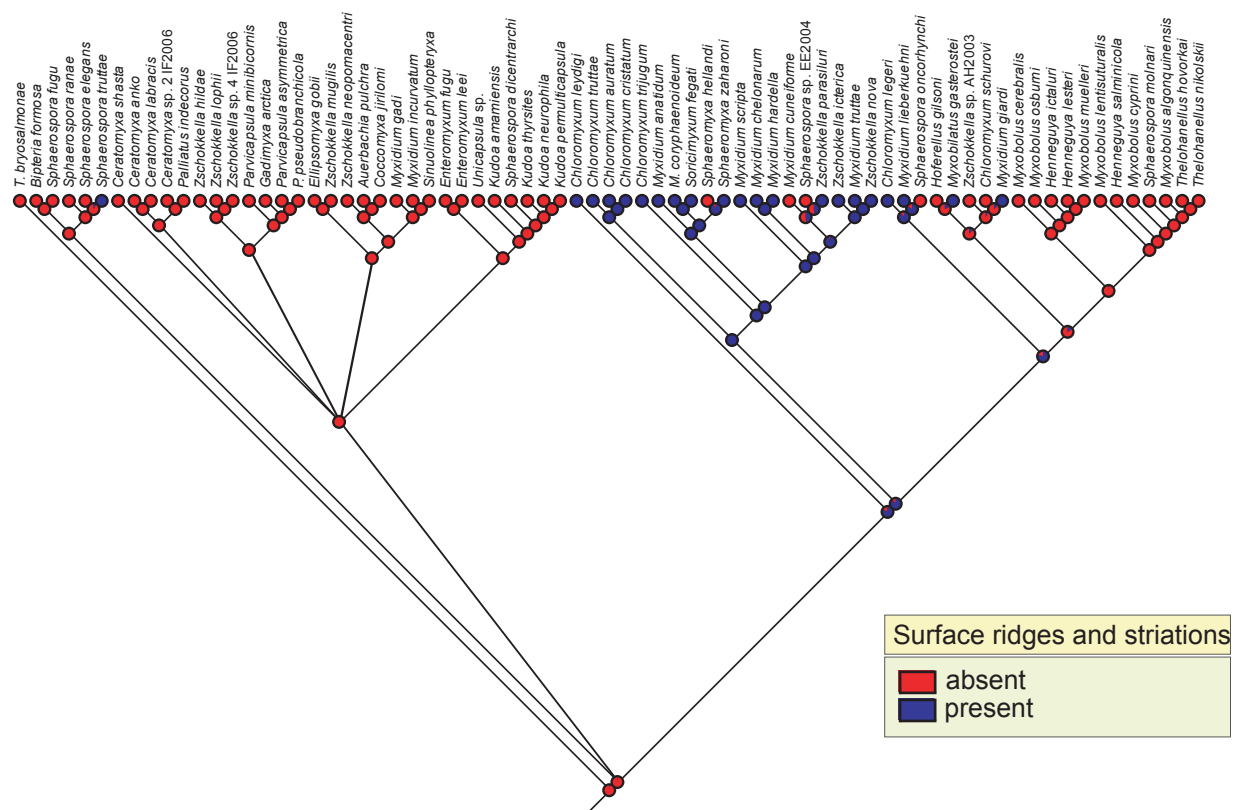

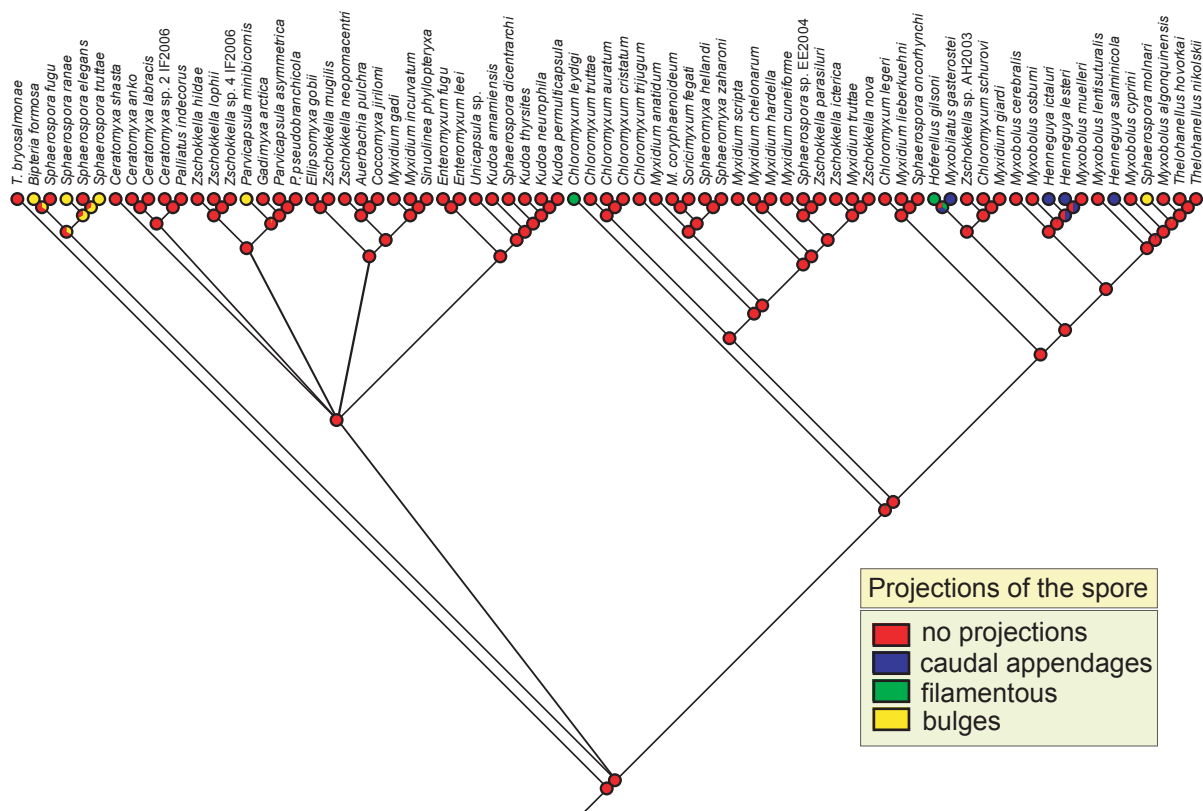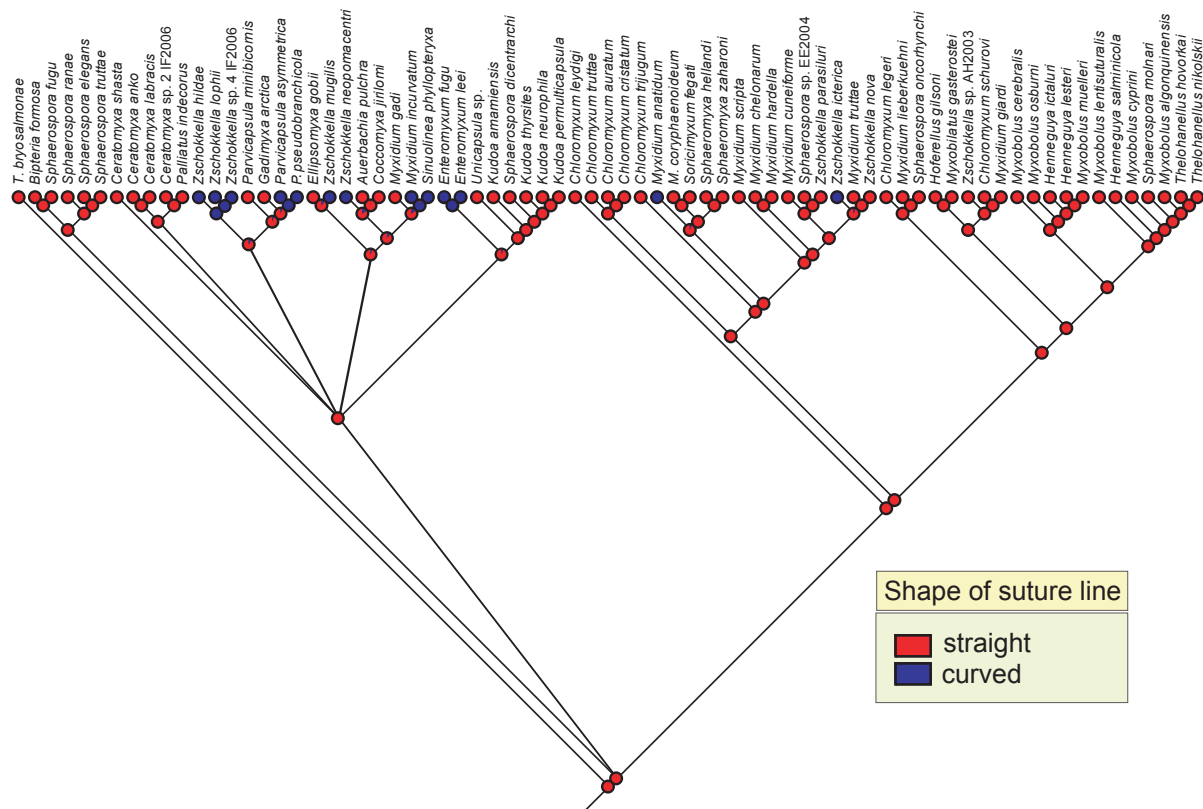

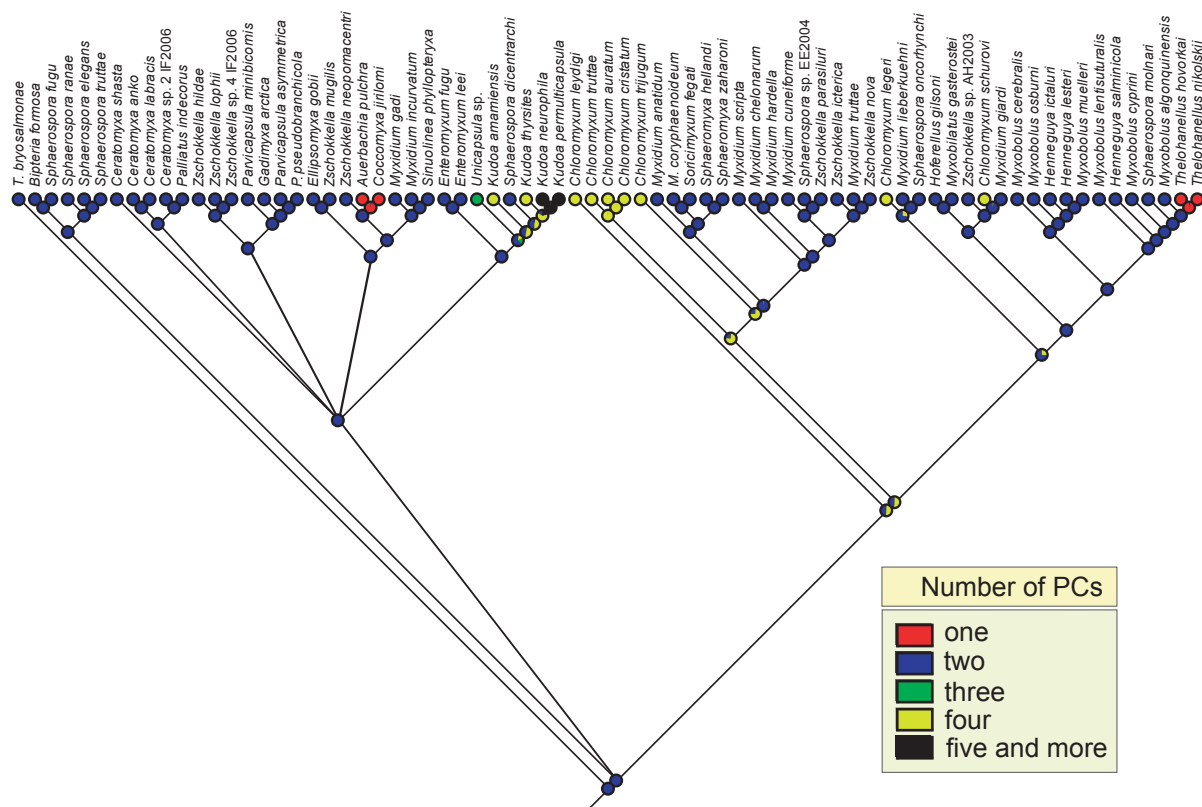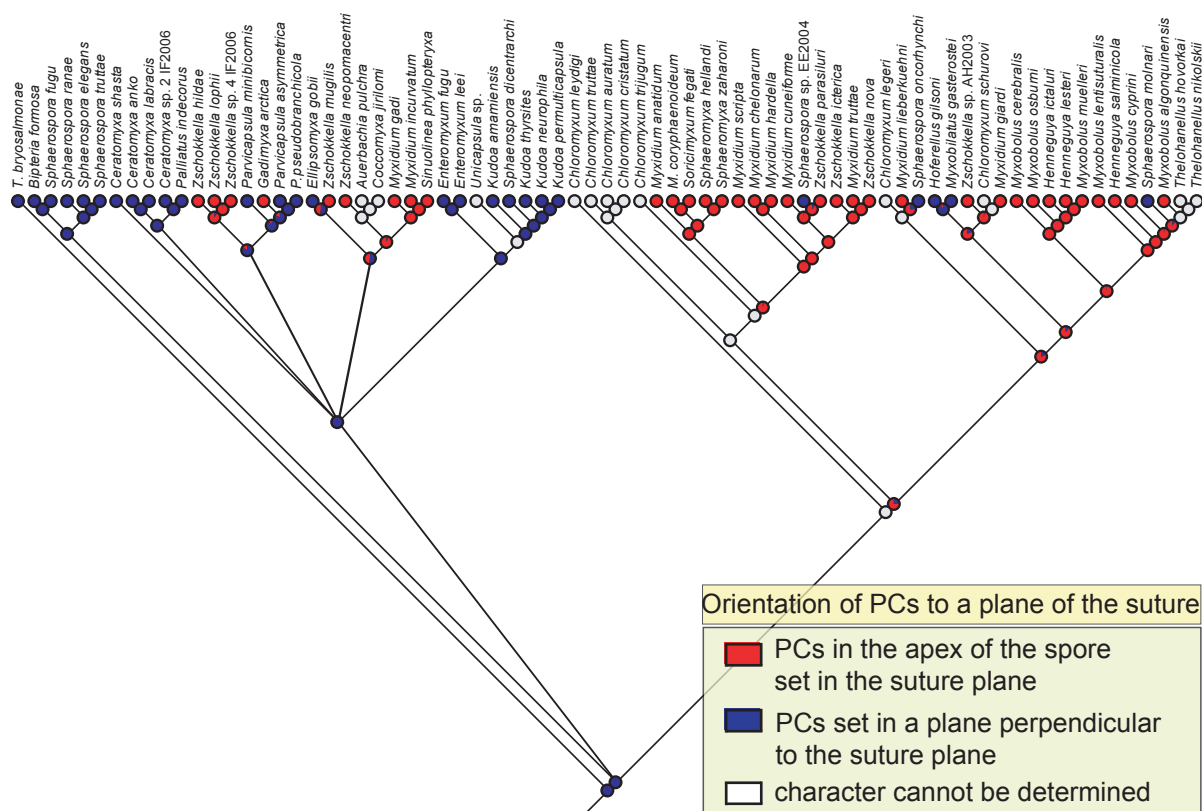

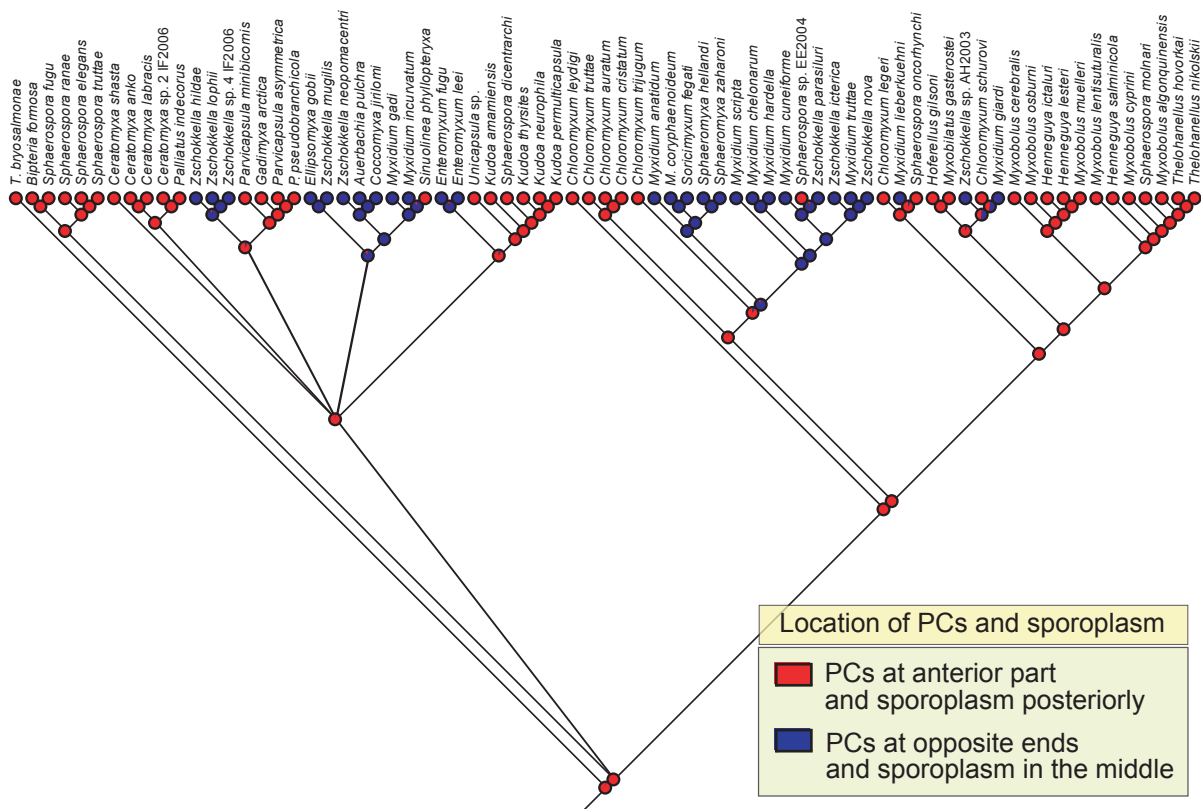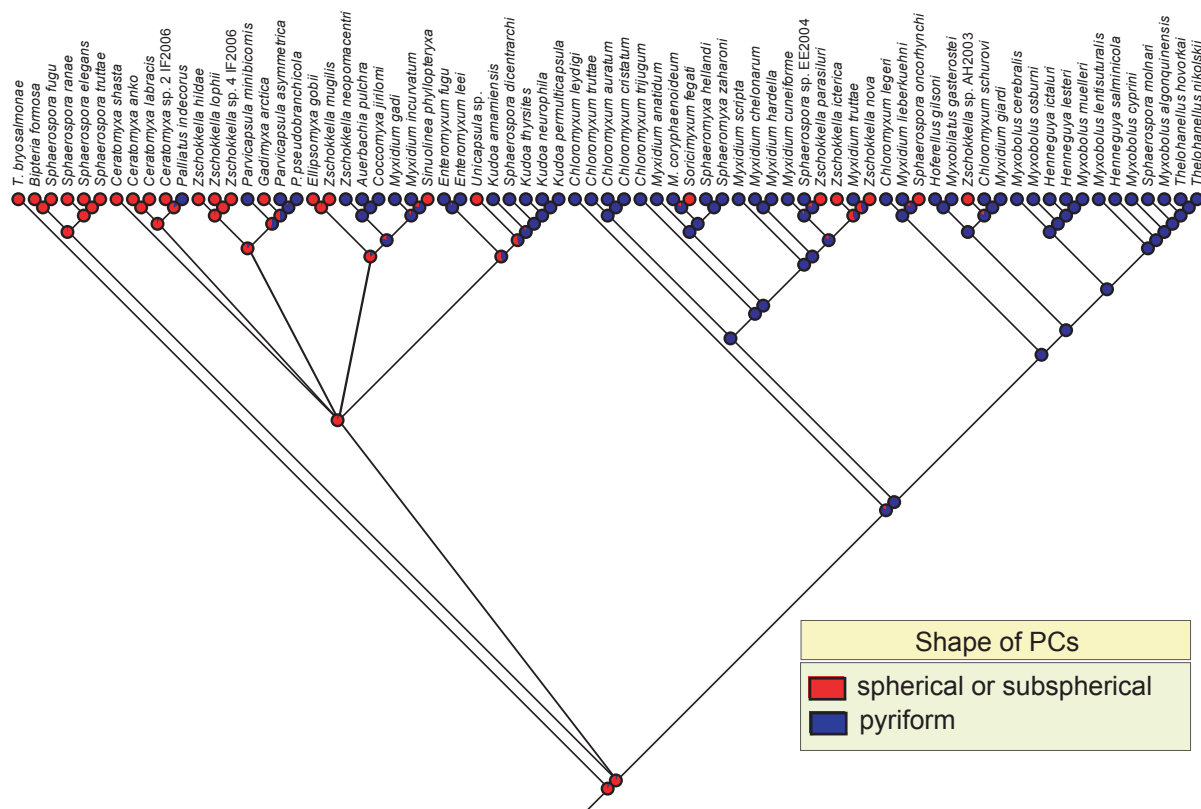

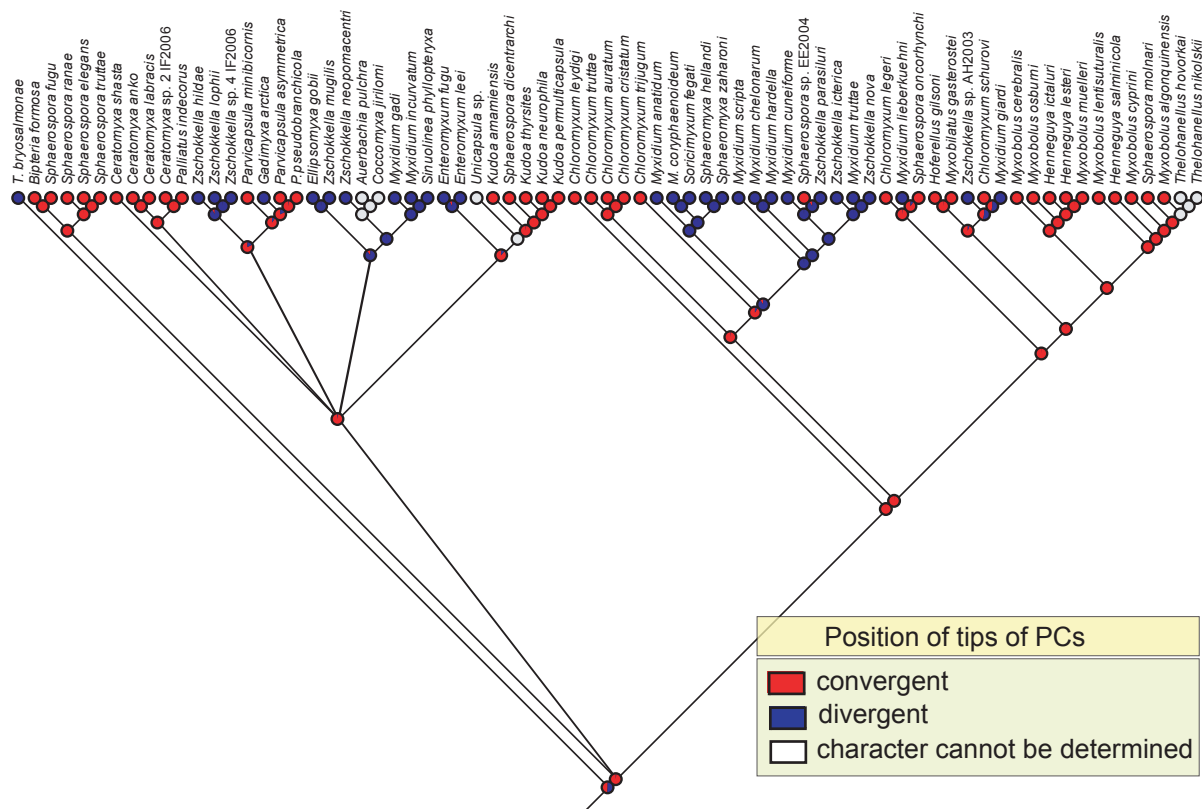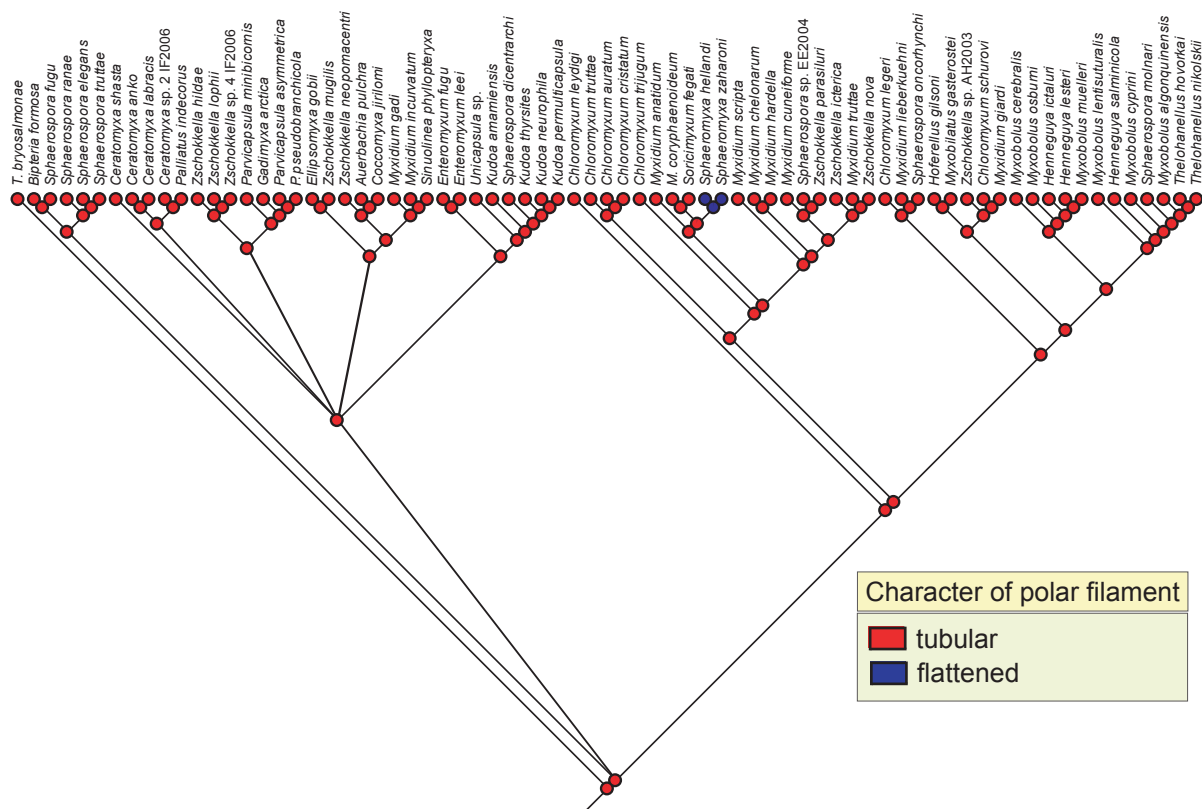

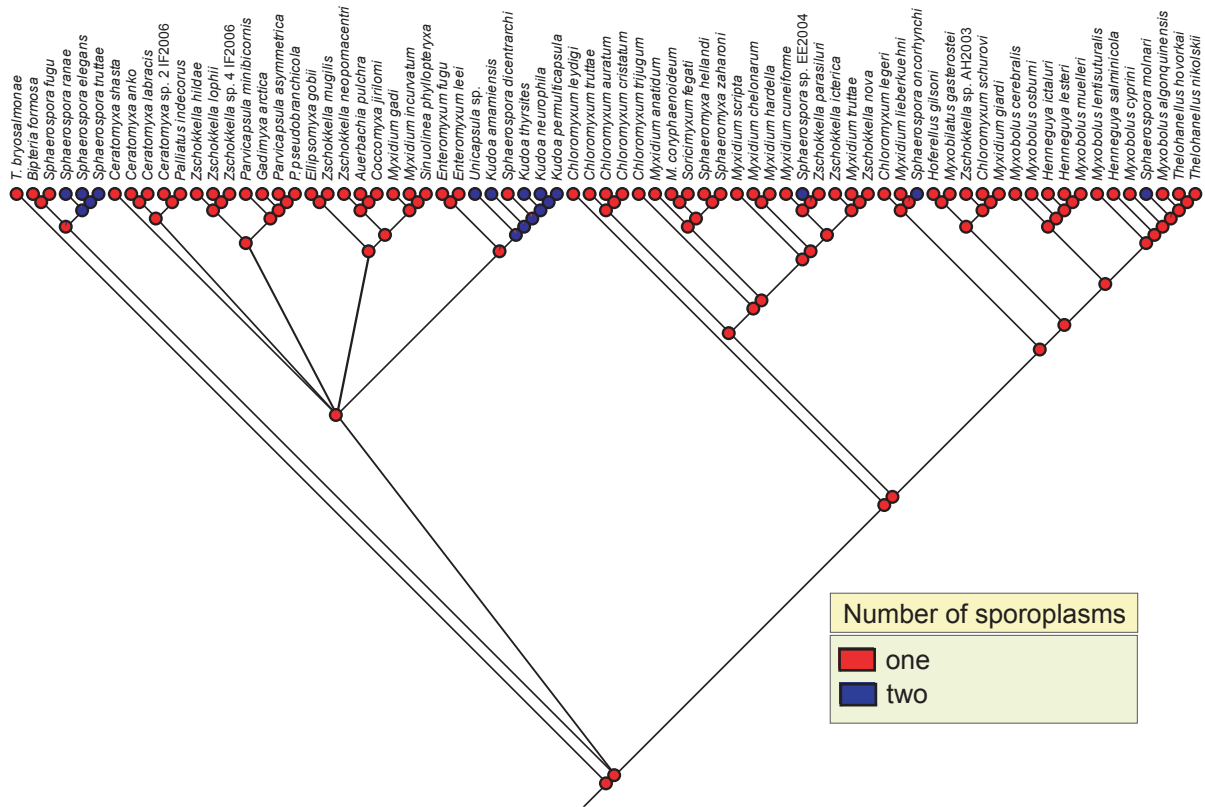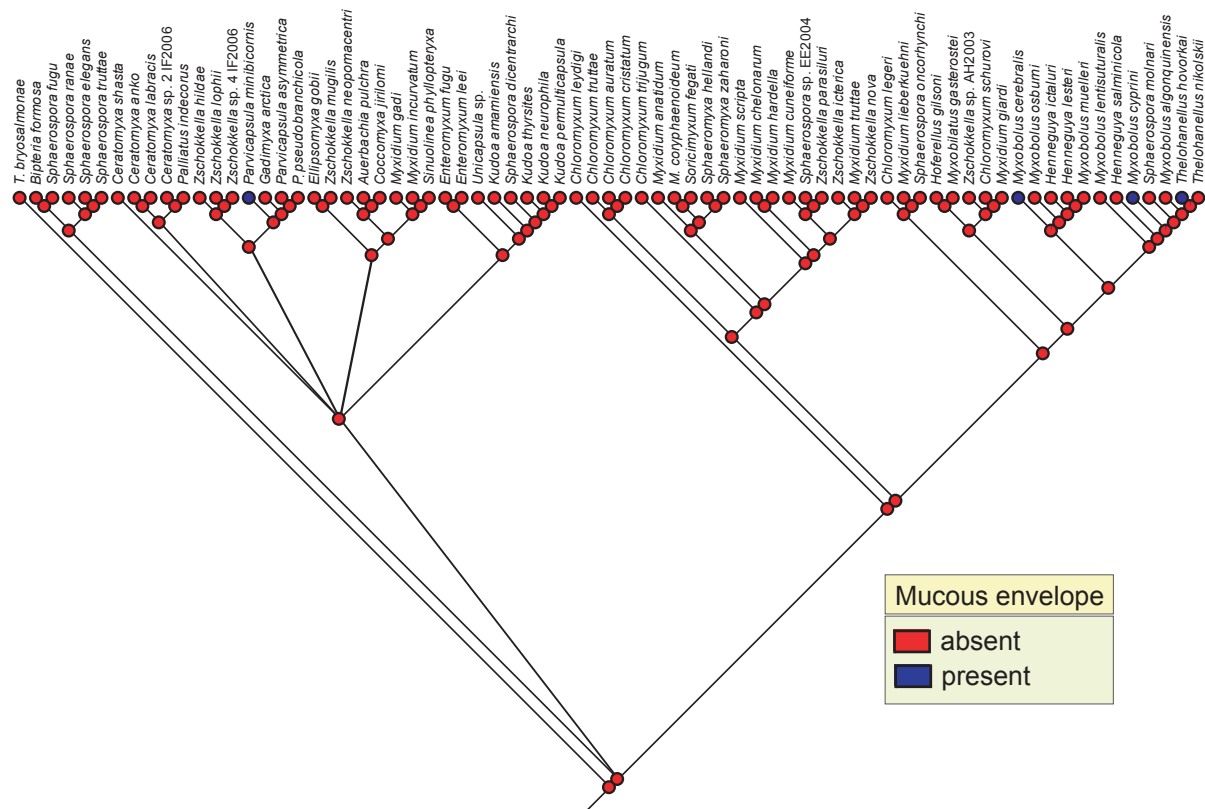

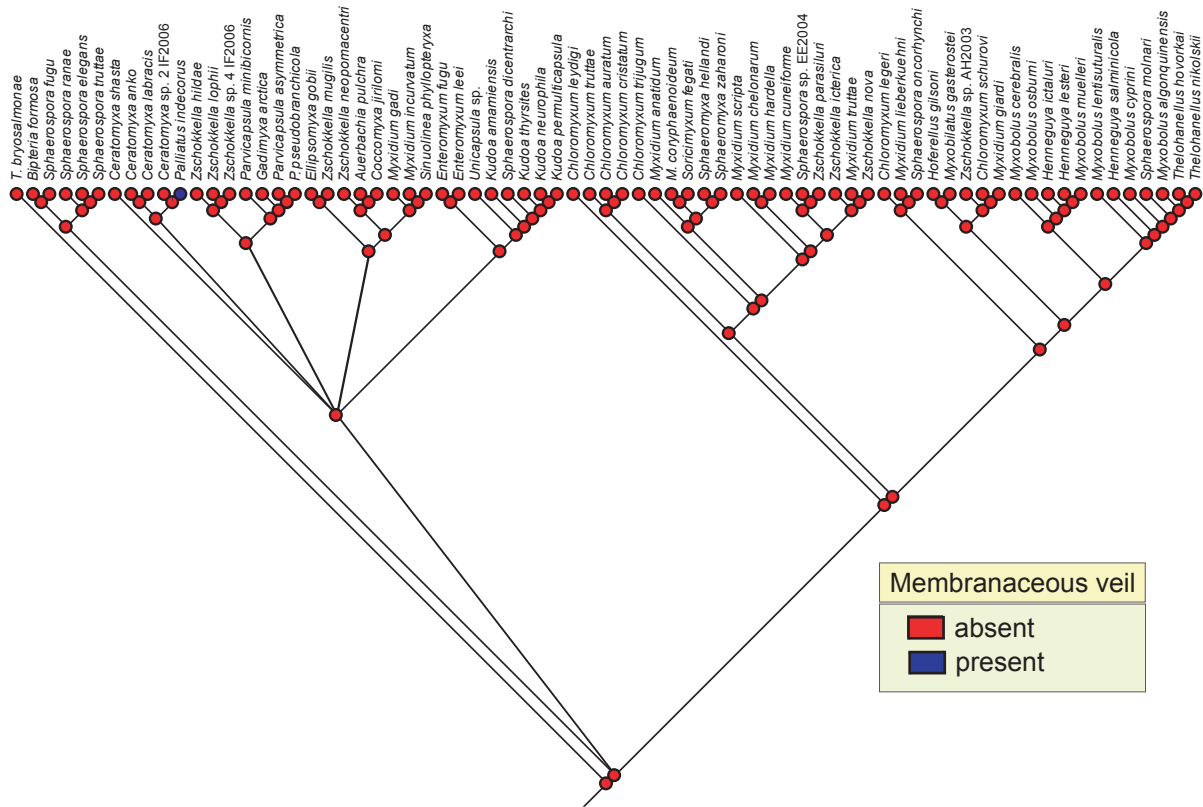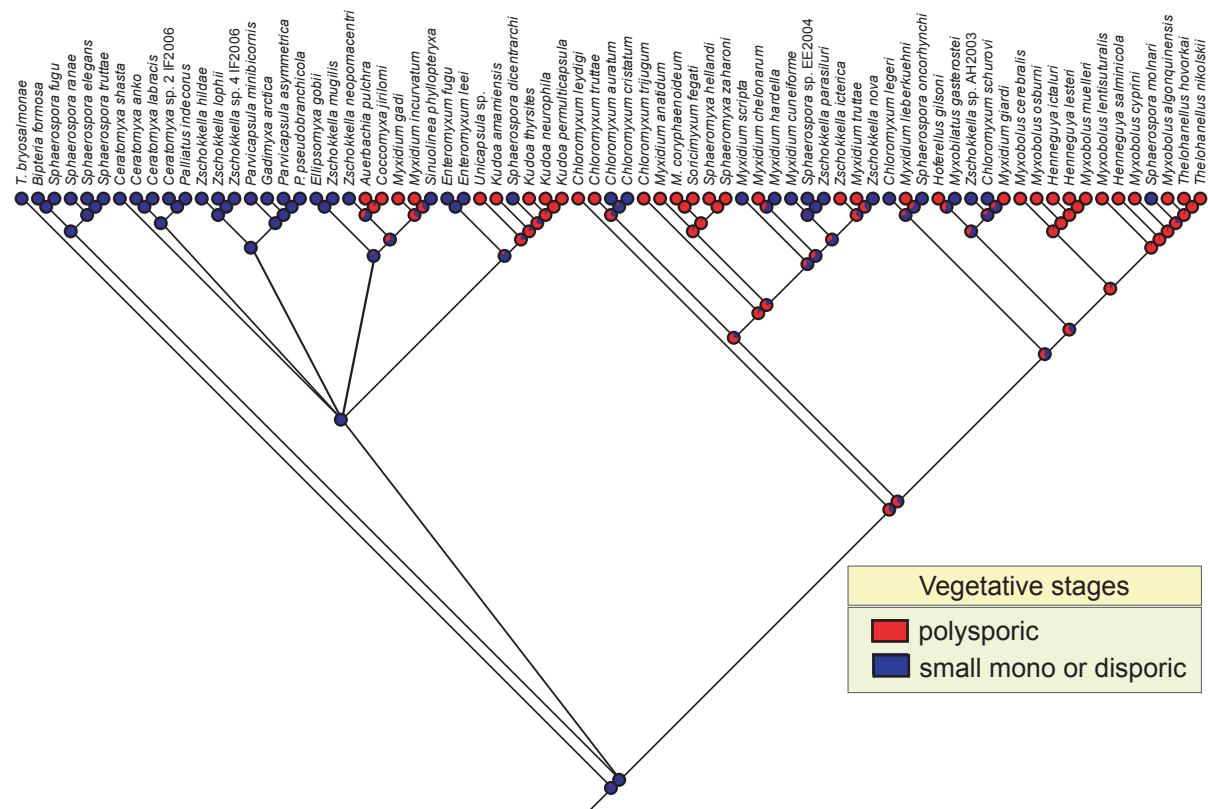

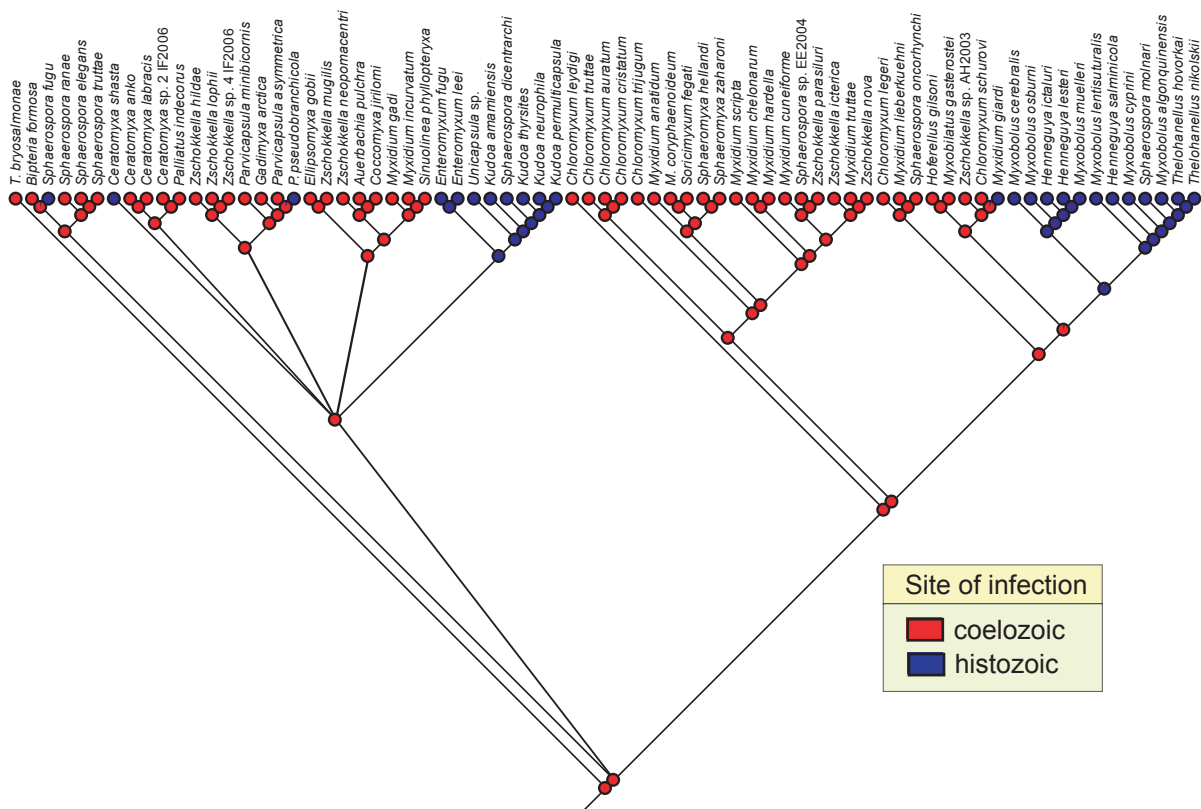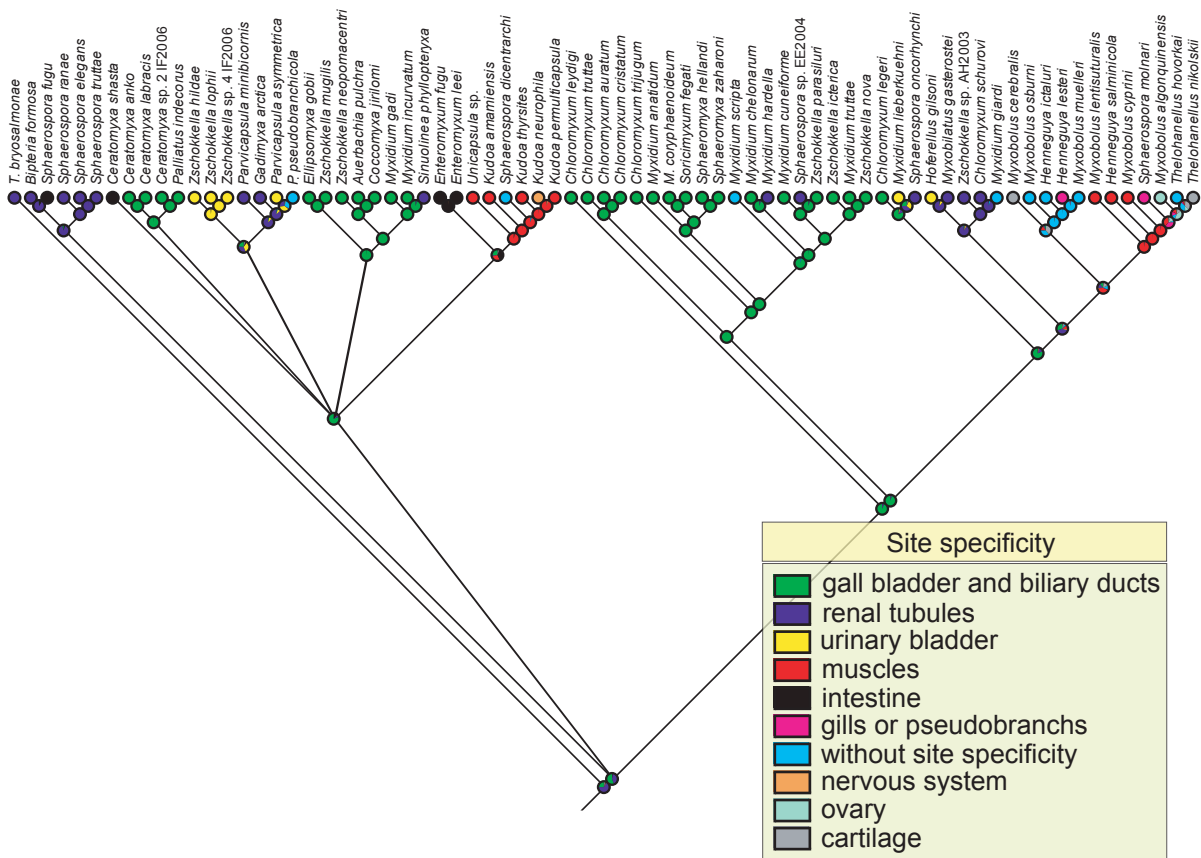

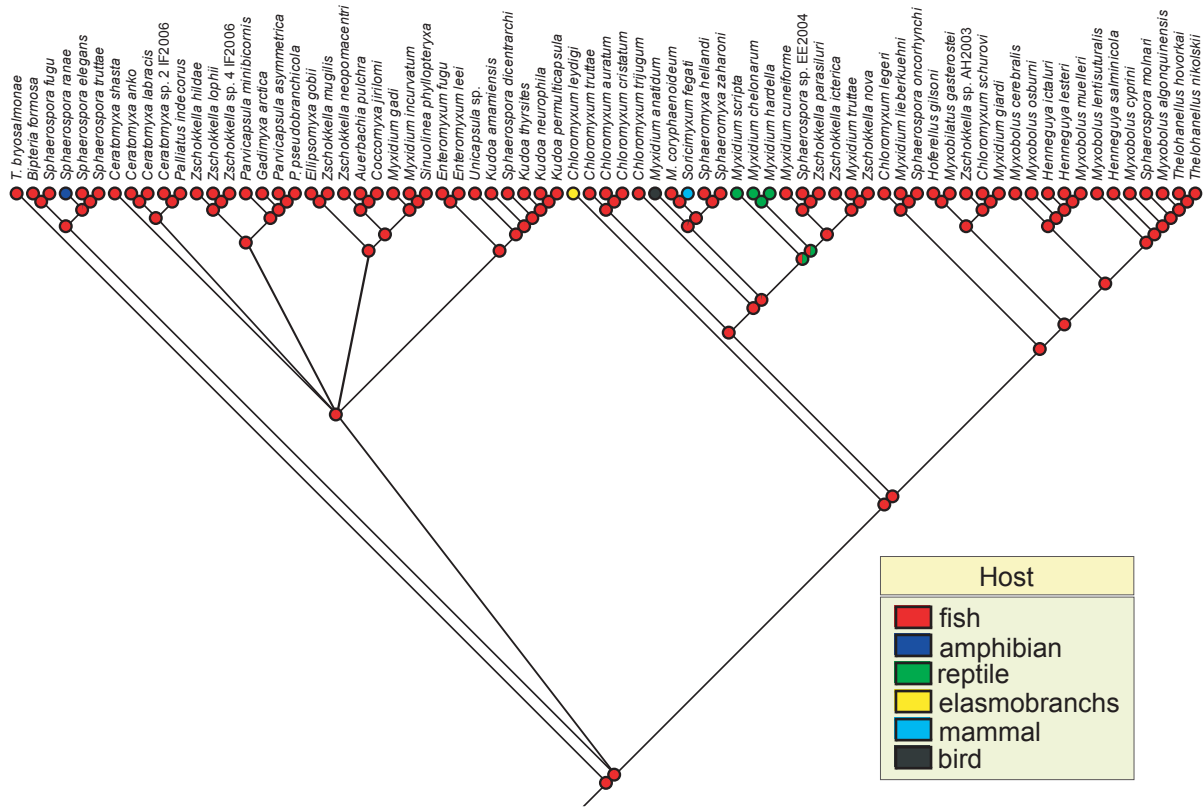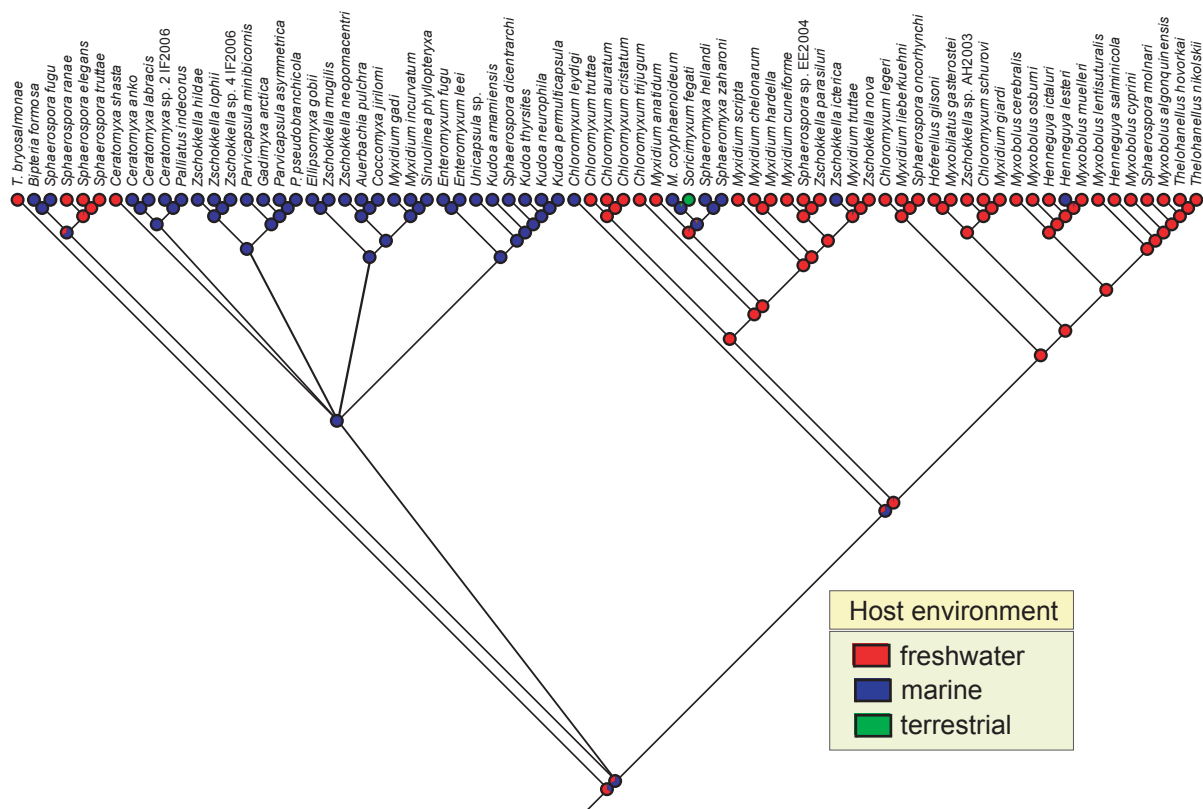

Supplement: Additional file 4 — Evolution of particular myxozoan characters. Twenty cladograms show the evolutionary history of all morphological and bionomical characters under study. The balls by the nodes represent proportional likelihoods of character states. See legends for colours of particular character states. [file 1471-2148-10-228-S4.PDF]
